# A novel fluorescence-based biosynthetic trafficking method provides pharmacologic evidence that PI4-kinase IIIα is important for protein trafficking from the endoplasmic reticulum to the plasma membrane

**DOI:** 10.1186/s12860-015-0049-5

**Published:** 2015-02-27

**Authors:** Kirsten L Bryant, Barbara Baird, David Holowka

**Affiliations:** Department of Chemistry and Chemical Biology, Cornell University, Ithaca, NY USA; University of North Carolina, Chapel Hill, NC 27514 USA

**Keywords:** Biosynthetic protein trafficking, Phosphoinositide 4-phosphate, Flow cytometry

## Abstract

**Background:**

Biosynthetic trafficking of receptors and other membrane-associated proteins from the endoplasmic reticulum (ER) to the plasma membrane (PM) underlies the capacity of these proteins to participate in crucial cellular roles. Phosphoinositides have been shown to mediate distinct biological functions in cells, and phosphatidylinositol 4-phosphate (PI4P), in particular, has emerged as a key regulator of biosynthetic trafficking.

**Results:**

To investigate the source of PI4P that orchestrates trafficking events, we developed a novel flow cytometry based method to monitor biosynthetic trafficking of transiently transfected proteins. We demonstrated that our method can be used to assess the trafficking of both type-1 transmembrane and GPI-linked proteins, and that it can accurately monitor the pharmacological disruption of biosynthetic trafficking with brefeldin A, a well-documented inhibitor of early biosynthetic trafficking. Furthermore, utilizing our newly developed method, we applied pharmacological inhibition of different isoforms of PI 4-kinase to reveal a role for a distinct pool of PI4P, synthesized by PI4KIIIα, in ER-to-PM trafficking.

**Conclusions:**

Taken together, these findings provide evidence that a specific pool of PI4P plays a role in biosynthetic trafficking of two different classes of proteins from the ER to the Golgi complex. Furthermore, our simple, flow cytometry-based biosynthetic trafficking assay can be widely applied to the study of multiple classes of proteins and varied pharmacological and genetic perturbations.

## Background

Studies of the phosphorylated derivatives of phosphatidylinositol (PI) have shown that these molecules possess distinct biological functions and localize selectively to organelles (reviewed by [1]). Due to variable phosphorylation of hydroxyl groups on their inositol rings, seven different inter-convertible phosphoinositide species exist in cells, including PI4P and phosphatidylinositol 4,5-bisphosphate (PI(4,5)P_2_). Different phosphoinositide species are often enriched in distinct intracellular membranes; for example, PI(4,5)P_2_ is predominately localized to the inner leaflet of the PM, whereas PI4P is enriched at the Golgi complex.

PI(4,5)P_2_ is a well-established regulator of multiple cellular processes, including vesicle trafficking [2], phagocytosis [3], membrane ruffling [4], cell motility and adhesion [5], and regulation of ion channel activity and receptor phosphorylation [6,7]. In addition, PI(4,5)P_2_ is the substrate for generation of the second messengers inositol 1,4,5-trisphosphate (IP_3_) and diacylglycerol (DAG) [8], and thereby is necessary for agonist-stimulated Ca^2+^ signaling. Furthermore, there have been several reports of PI(4,5)P_2_ existing in functionally and spatially distinct pools in the PM that support specific signaling platforms [9-12]. PI4P, the most prevalent mono-phosphorylated PI-derivative in cells [13], was for many years believed to serve no function outside of being the precursor for PIP_2_ [14]. Recently, however, a number of PI4P-dependent processes have been characterized, in particular its role in the regulation of protein trafficking. For example, PI4P strongly promotes COPII-mediated export of proteins at endoplasmic reticulum (ER) exit sites (ERES; [15,16]). Also, by interacting with the lipid transfer proteins CERT, OSBP, and FAPP (collectively termed COFs), PI4P plays roles in sphingolipid and sterol biosynthetic trafficking [17].

Organelle-specific phosphoinositide distributions are maintained by the tight regulation of PI-kinases and PI-phosphatases. Four distinct PI 4-kinases have been described in mammalian cells, including type II (PI4KIIα and PI4KIIβ) and type III (PI4KIIIα and PI4KIIIβ) kinases [18]. The type II PI 4-kinases are palmitoylated [19] and thus strongly membrane associated, particularly in the trans-Golgi apparatus [20], and, to a lesser extent, in endosomes [21]. PI4KIIIβ localizes primarily to the Golgi apparatus, coincident with Arf1, a small GTP-binding protein [22,23]. Although the molecular details of how these enzymes are linked to Golgi-derived biosynthetic transport remain unknown, they have all been implicated in Golgi function and secretion [21]. Deletion of the gene for PI4KIIIα is embryonically lethal in mice [24], and its normal subcellular distribution is complex, with evidence for cytosolic [24], PM [24,25], and ER [22] concentration. Recently, PI4KIIIα has been identified as a critical host factor for hepatitis C viral replication [26]. With regard to biosynthetic trafficking, PI4P localized to the Golgi apparatus has been implicated in the delivery of cargo from the Golgi to the PM [27,28], in addition to evidence supporting a role for PI4P in COPII nucleation at ERES cited above [15,16].

The present study addresses PI4P participation in ER-to-Golgi trafficking. Using a novel technique to monitor protein biosynthetic trafficking, we show that pharmacological inhibition of PI4KIIIα results in ER-retention of both the epidermal growth factor receptor (EGFR) and a glycophosphatidylinositol (GPI)-anchored protein. Furthermore, we show that inhibition of a Golgi-localized PI 4-kinase does not result in ER-retention. Taken together, these findings provide evidence that a specific pool of PI4P, synthesized by PI4KIIIα, is essential for biosynthetic protein trafficking.

## Results

### Flow cytometry of transiently transfected cells to assess perturbations of early ER-to-Golgi trafficking events

**Figure 1:**
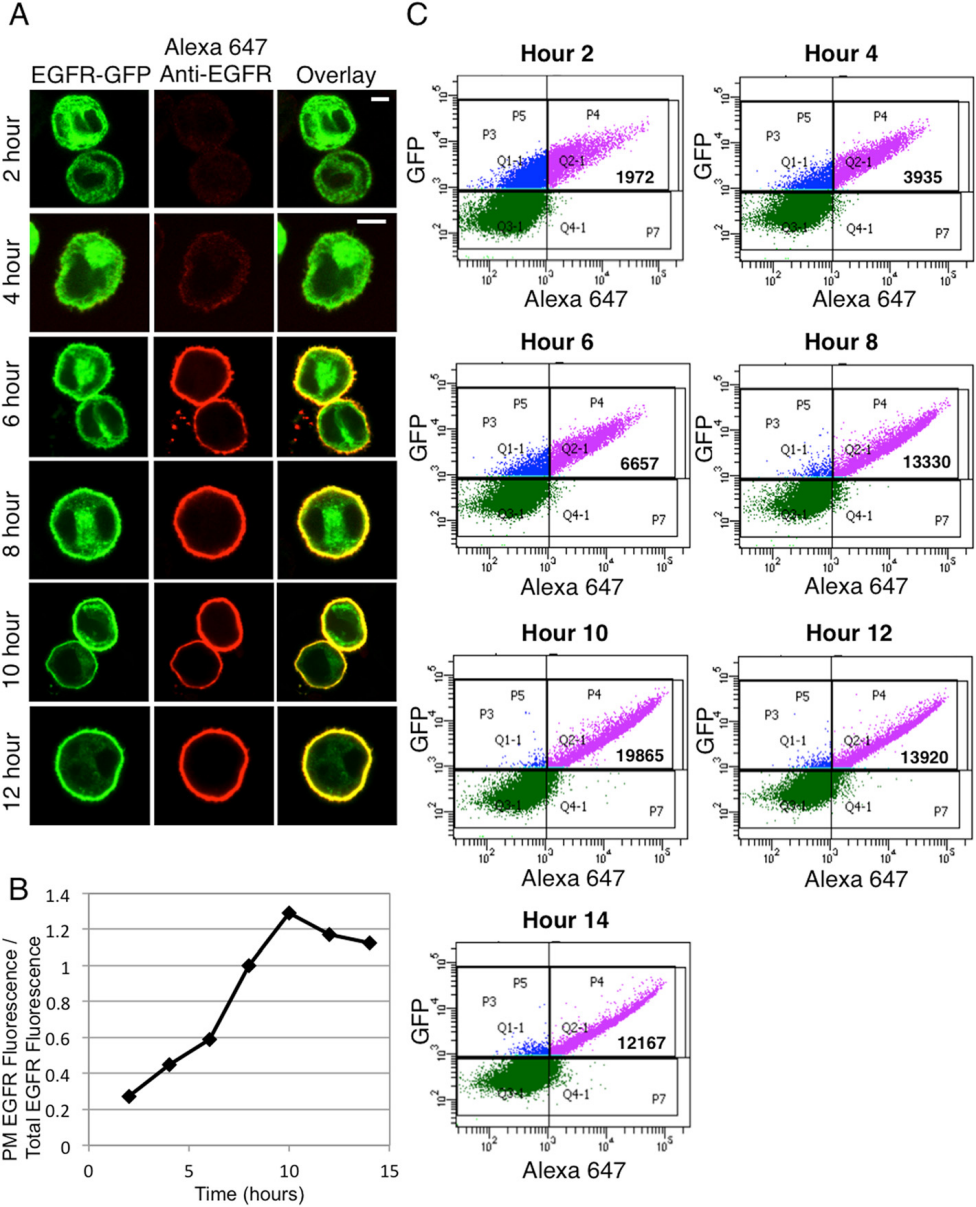
**Robust trafficking of EGFR to the PM upon transferring expressing cells to 37°C following 14 hrs at 22°C.** Live cells transiently expressing EGFR-GFP were harvested for flow cytometry, then fixed at each time point. Cells were incubated with an anti-N-terminal EGFR antibody followed by Alexa647-anti-IgG to label PM-associated EGFR, then imaged by confocal microscopy **(A)** or analyzed by flow cytometry **(B)** to determine the ratio of PM-localized EGFR fluorescence to total-EGFR fluorescence. Plot is representative of typical results; approximately 8,000 EGFR-GFP expressing cells were analyzed at each time point, and the ratios for all time points were normalized to that at 8 hr. Scale bars in **A** are 5 μm. **(C)** Representative flow cytometric scatter plots. RBL-2H3 cells transiently expressing GFP-tagged EGFR constructs were harvested, fixed, and labeled with anti-N-terminal EGFR antibody followed by Alexa 647-anti-IgG. Cells were gated on positive GFP fluorescence (blue and purple populations), and Alexa 647 fluorescence was analyzed to determine PM localization (purple population). Bold numbers indicate the mean Alexa 647 fluorescence of the GFP-positive population. Plots are representative of data used to generate “control” traces in Figures 1, 2, 4, and 7.

Preliminary experiments indicated that testing the effects of potential inhibitors of ER-to-Golgi trafficking requires a method in which a substantial percentage of transfected protein makes this transition simultaneously. To synchronize the early stages of biosynthetic trafficking, we developed a protocol in which RBL-2H3 mast cells are incubated at 22°C for 14 hr following transient transfection with EGFR-GFP. This incubation at lower temperature allows for protein synthesis to occur with minimal trafficking to the PM [29,30]. The next day, the cells are transferred to 37°C, at which time biosynthetic trafficking begins. Cells are harvested and fixed at the various time points (depending on the experiment), and unpermeabilized cells are labeled with an antibody specific for an extracellular epitope. Thus, the increased presence of EGFR-GFP at the PM can be quantified over time by flow cytometry by measuring the total EGFR-GFP fluorescence and comparing it to the fluorescence of the extracellular epitope-labeling antibody. Representative confocal images of the suspended flow cytometry samples show that 6 hr after transfer to 37°C, EGFR is clearly detectable at the PM (Figure 1A). In agreement with previous reports on the timescale of EGFR protein maturation [31], most cells show predominantly ER-localized EGFR at 2 hr after transfer, with no detectable labeling at the PM, and by 4 hr significant Golgi localization is observed, with some receptor localization at the PM (Figure 1A). Under these conditions, trafficking of EGFR-GFP to the PM is maximal by 10 hr at 37°C, as quantified by flow cytometry (Figure 1B,C).

**Figure 2:**
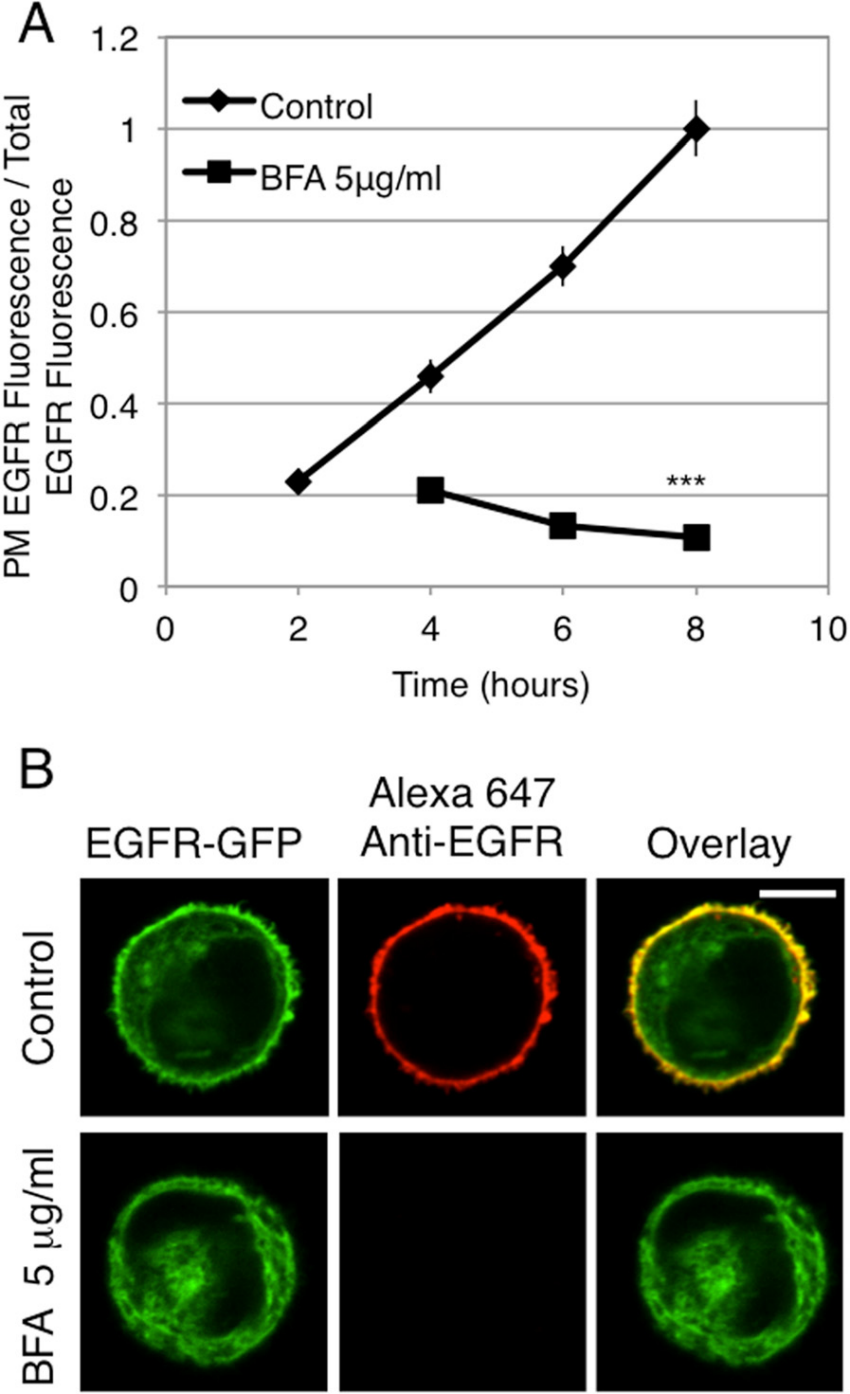
**Treatment with brefeldin A inhibits the biosynthetic trafficking of EGFR to the PM.** Live cells transiently expressing EGFR-GFP were processed as described for Figure 1, except that BFA (5 μg/ml) was added to half of the cells at hour 3. **(A)** Flow cytometry was used to calculate the ratio of PM localized EGFR fluorescence to total EGFR fluorescence, and this ratio for each time point ± BFA is plotted. Error bars indicate ± SE of four independent experiments in which approximately 8,000 EGFR-GFP expressing cells were analyzed at each time point. *** p < 0.001 **(B)** Confocal images of RBL cells expressing EGFR-GFP either untreated or treated with BFA, cells are from 8 hr time points quantified in **A**. Scale bars show 5 μm.

**Figure 3:**
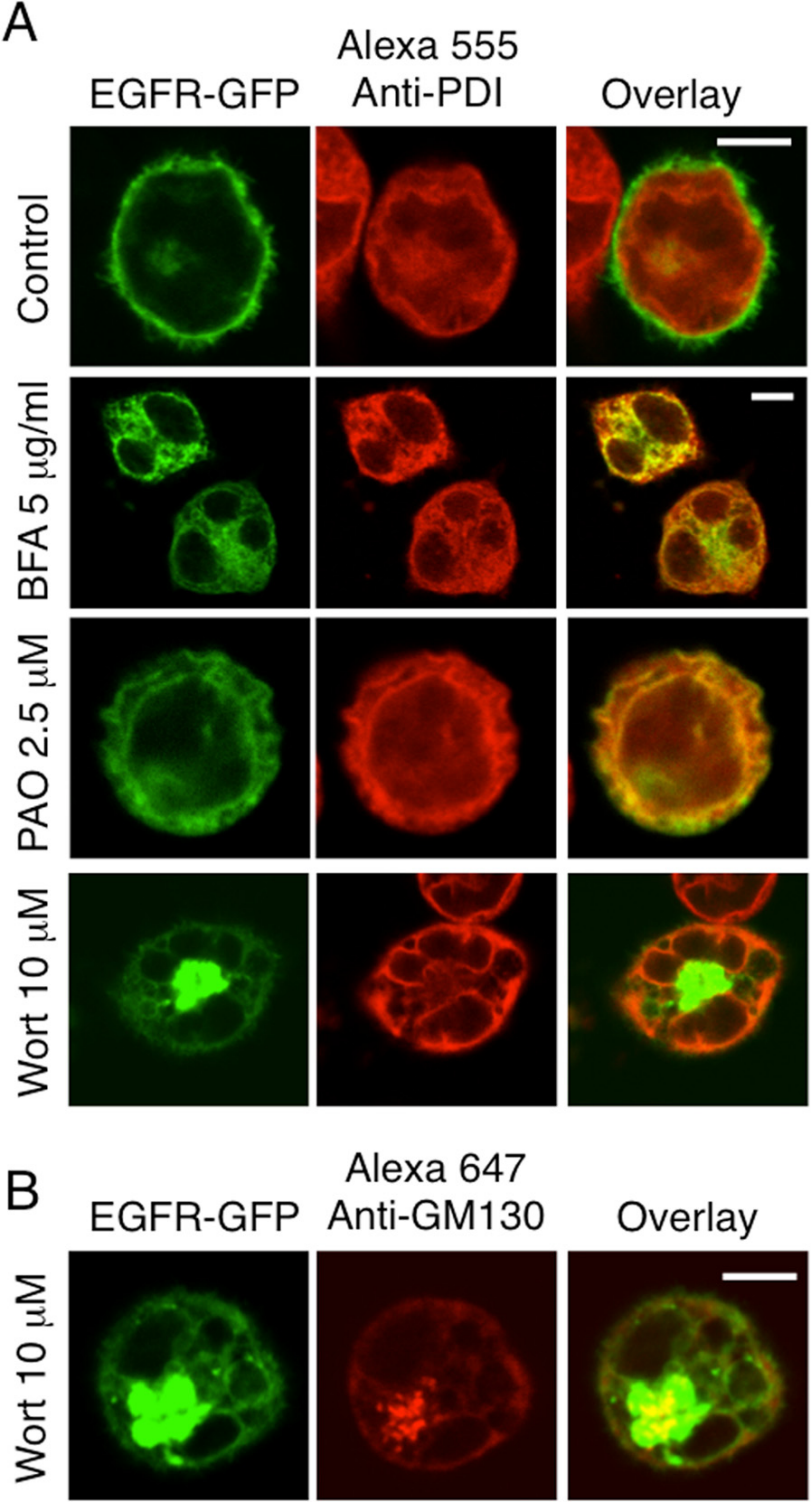
**EGFR-GFP is ER-retained in cells treated with BFA (5 μg/ml) and PAO (2.5 μM), whereas in wortmannin (10 μM) treated cells, retention occurs in the ER and Golgi apparatus. (A)** Attached RBL cells transiently expressing EGFR-GFP were processed as described for Figure 1, except that inhibitors were added at hour 3. At hour 8 cells were fixed, permeabilized, and incubated with an anti-PDI antibody followed by Alexa 555-anti-mouse IgG to label the ER lumen. **(B)** The same cells shown in **A** were incubated with an anti-GM130 antibody followed by Alexa 647-anti-mouse IgG to label the Golgi apparatus. Scale bars show 5 μm.

To determine whether we could accurately monitor the pharmacological disruption of biosynthetic trafficking using this method, we first tested the effects of brefeldin A (BFA), a well-documented inhibitor of early biosynthetic trafficking [32]. Because we observed that EGFR transfers from ER to Golgi by 4 hr after the temperature shift (Figure 1A), we chose to apply the inhibitor at 3 hr. When RBL cells expressing EGFR-GFP are treated with 5 μg/ml BFA under these conditions, EGFR-GFP trafficking to the PM is prevented (Figure 2A), and EGFR-GFP exhibits an intracellular distribution (Figure 2B). Evidence that EGFR-GFP is retained in the ER in the presence of BFA is provided by immunofluorescence co-localization with the resident lumenal ER protein, PDI (Figure 3A).

### Inhibition of PI4KIIIα by PAO prevents EGFR trafficking from the ER to the Golgi apparatus

**Figure 4:**
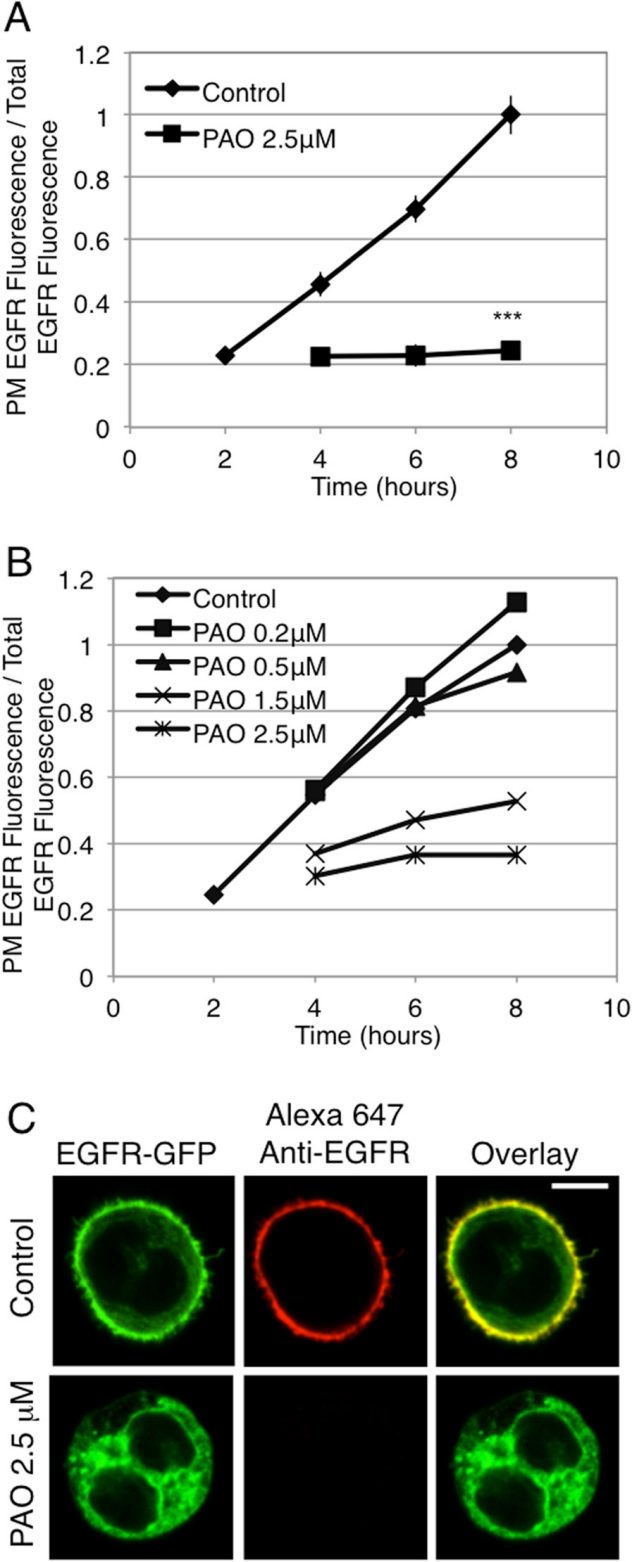
**Treatment with PAO inhibits the biosynthetic trafficking of EGFR to the PM in a dose-dependent manner** ***.*** Live cells transiently expressing EGFR-GFP were processed as described for Figure 1, except that PAO was added at indicated concentrations to half of the cells at hour 3. **(A)** Flow cytometry was used to calculate the ratio of PM localized EGFR fluorescence to total EGFR fluorescence, and this ratio for each time point ± PAO (2.5 μM) is plotted. Error bars indicate ± SE of five independent experiments in which approximately 8,000 EGFR-GFP expressing cells were analyzed at each time point. *** p < 0.001 **(B)** Dose-dependence of PAO inhibition of biosynthetic trafficking of EGFR. Samples were processed as in **A** following addition of indicated concentrations of PAO. Data from one experiment is representative of 2 separate experiments; approximately 8,000 EGFR-GFP expressing cells were analyzed at each time point. **(C)** Confocal images of RBL cells expressing EGFR-GFP either untreated or treated with PAO, cells are from 8 hr time points quantified in **A**. Scale bar shows 5 μm.

**Figure 5:**
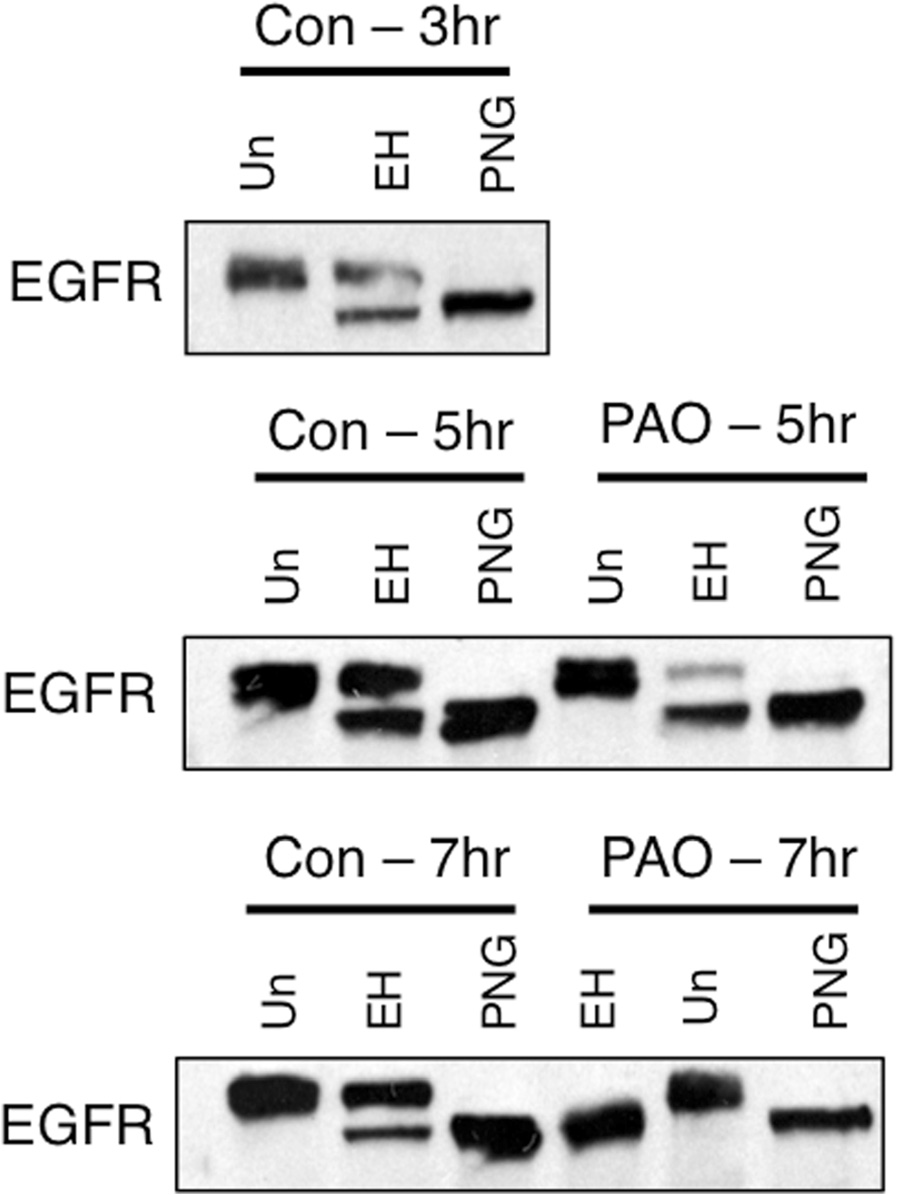
**Analysis of biosynthetic trafficking by susceptibility to digestion by endoglycosidase H.** RBL mast cells transiently expressing pkH3-EGFR were subjected to the same trafficking protocol as described in Figure 1. However, rather than processing samples for flow cytometry, whole cell lysates were collected and were either untreated (Un), treated with EndoH (EH), or treated with PNGase F (PNG) for 24 hr before Western blot analysis with an anti-EGFR antibody. The EGFR protein from the PAO treated cells is completely Endo H sensitive by 7 hr, thus supporting the contention that these EGFR proteins contain only N-linked oligosaccharide chains and have not trafficked beyond the ER.

Based on previous evidence for a role for PI4P in COPII nucleation at ERES [15,16], we hypothesized that a pool of PI4P generated by PI4KIIIα is necessary for proper biosynthetic trafficking from the ER to the Golgi apparatus. Phenylarsine oxide (PAO) is an inhibitor of PI 4-kinases [33], which, when present in low micromolar concentrations, selectively inhibits the PI4KIIIα isoform [18,21]. We recently showed that PAO inhibits IgE receptor signaling responses that are downstream of stimulated tyrosine phosphorylation in RBL mast cells by a mechanism consistent with inhibition of phosphoinositide synthesis [34]. Under these conditions, acute addition of PAO does not inhibit IgE receptor-mediated tyrosine phosphorylation or thapsigargin-stimulated Ca^2+^ mobilization. When RBL cells expressing EGFR-GFP are treated with 2.5 μM PAO, trafficking of these receptors to the PM is prevented (Figure 4A), and concentrations as low as 1.5 μM substantially inhibit biosynthetic trafficking (Figure 4B). In the presence of 2.5 μM PAO, EGFR-GFP is retained intracellularly (Figure 4C) and is co-localized with PDI throughout the ER (Figure 3A). When biosynthetic trafficking out of the ER is assessed by susceptibility to digestion by endoglycosidase H (EndoH), we find that 2.5 μM PAO substantially inhibits the appearance of an EndoH-resistant pool of EGFR (Figure 5). EndoH acts by cleaving N-linked oligosaccharide chains near their site of attachment to the protein, and, once a protein has trafficked to the Golgi, further oligosaccharide processing results in resistance to EndoH digestion. Peptide-N-Glycosidase F (PNGase F) removes all glycosylations, and serves as a control for these assays. The appearance of an EndoH-resistant pool of EGFR in the 3 hr control sample in Figure 5 indicates that some exit from the ER has occurred by this time point, even though this is minimally detectable after 2 hr by flow cytometry and confocal microscopy (Figure 1).

### The requirement of PI4P synthesized by PI4KIIIα for ER-to-Golgi trafficking is not limited to EGFR or type-1 transmembrane proteins

**Figure 6:**
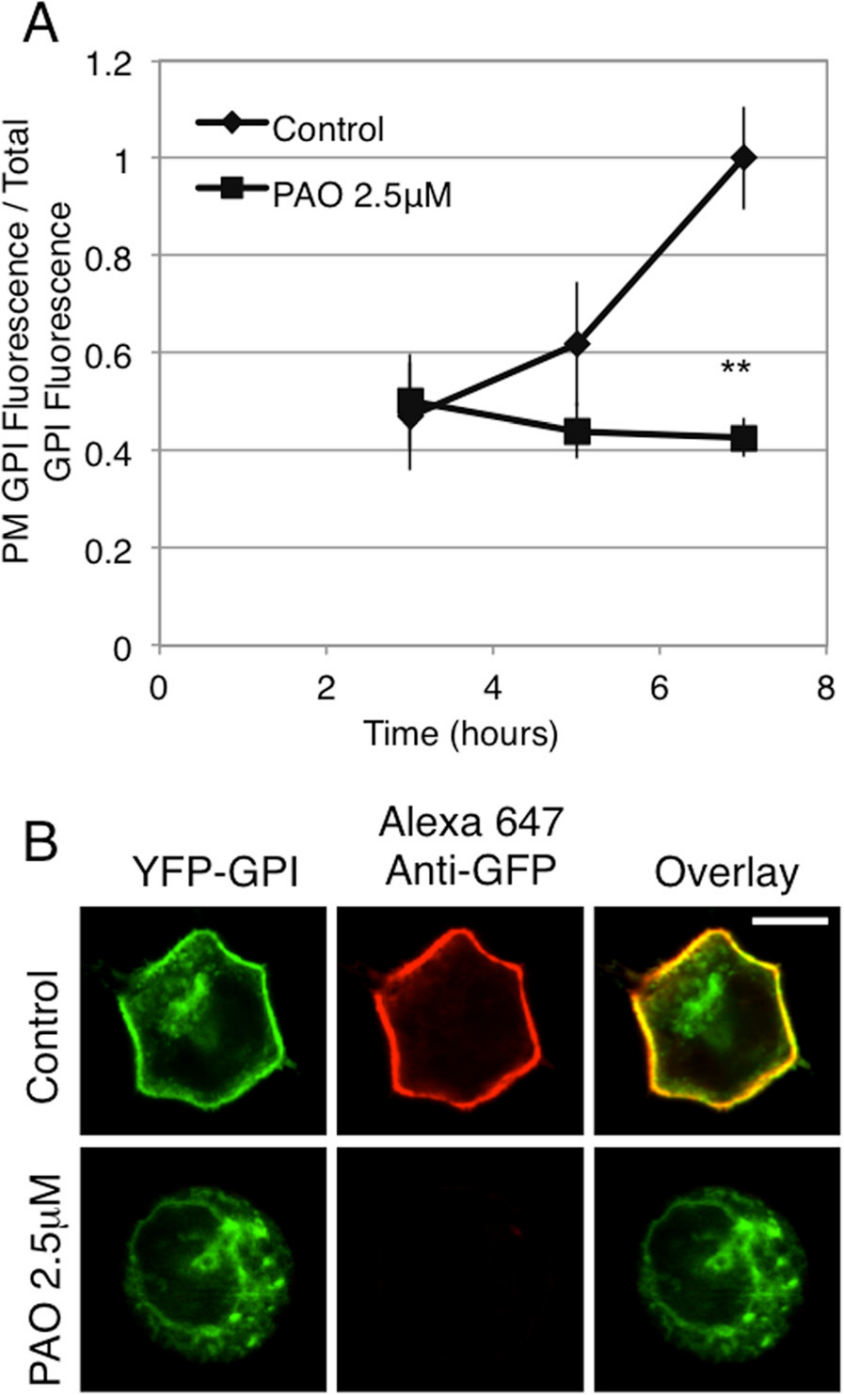
**Treatment with PAO inhibits the biosynthetic trafficking of GPI to the PM.** Live cells transiently expressing GPI-YFP were processed as described for EGFR expressing cells, PAO (2.5 μM) was added to half of the cells at hour 3. **(A)** Flow cytometry was used to calculate the ratio of PM localized GPI fluorescence, detected by anti-GFP, to total GPI fluorescence, and this ratio for each time point ± PAO (2.5 μM) is plotted. Error bars indicate ± SD of three independent experiments in which approximately 8,000 GPI-YFP expressing cells were analyzed at each time point, and all time points were normalized to the control ratio at 7 hr, ** p < 0.01. **(B)** Confocal images of attached RBL cells expressing GPI-YFP either untreated or treated with PAO for 6 hr, as quantified in **A**. Scale bar shows 5 μm.

The GPI anchor is a posttranslational modification that results in expression of modified proteins in the outer leaflet of the PM [35]. Synthesis of the GPI-linker begins on the cytoplasmic face of the ER; the precursor is then flipped to the lumenal side of the ER for further processing and protein attachment before trafficking to the PM [36]. We performed our biosynthetic trafficking protocol with a construct consisting of the GPI anchor of LFA-3, a heavily glycosylated surface protein of broad tissue distribution [37], fused to YFP. In the hours following the temperature shift, we observed increased PM expression of GPI-YFP, and this process was prevented by treating the cells with 2.5 μM PAO (Figure 6). These results suggest that PI4P synthesized by PI4KIIIα is involved in ER-to-PM trafficking of at least two different classes of proteins.

### Treatment with the PI4K inhibitors wortmannin and quercetin prevent the biosynthetic trafficking of EGFR to the PM

Although low micromolar concentrations of PAO selectively inhibit the type-IIIα isoform of PI 4-kinase, PAO can also inhibit tyrosine phosphatases at these concentrations [38]. To distinguish whether retention of EGFR in the ER upon PAO treatment is due to phosphatase or PI 4-kinase inhibition, we tested the effects of the PI 4-kinase inhibitors wortmannin and quercetin [2-(3,4-dihydroxy-phenyl)-3,5,7-trihydroxy-4*H*-chromen-4-one], neither of which inhibits tyrosine phosphatases at the concentrations used [34,39,40].

**Figure 7:**
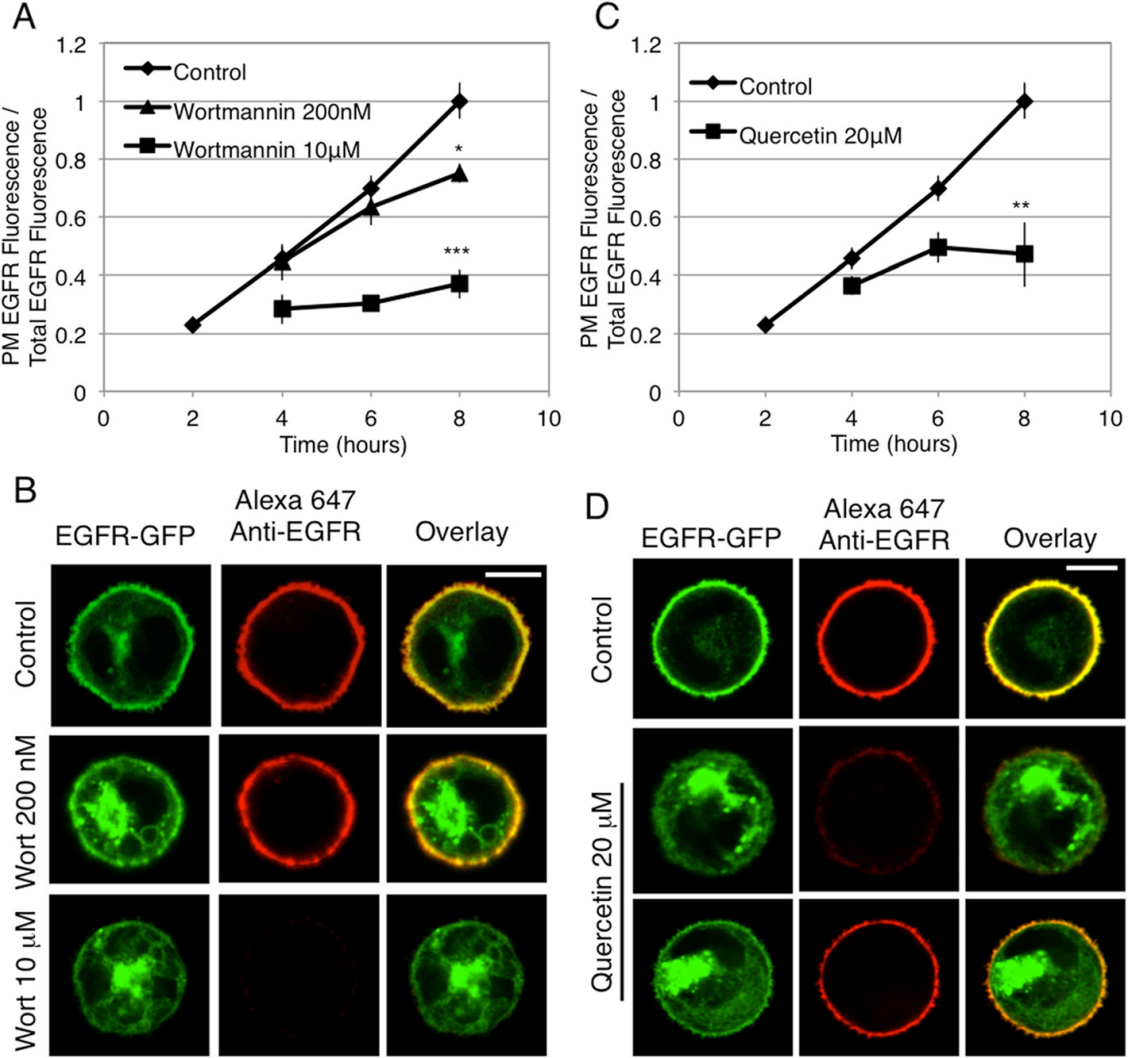
**Treatment with the PI4K inhibitors wortmannin and quercetin prevents the biosynthetic trafficking of EGFR to the PM.** Live cells transiently expressing EGFR-GFP were processed as described for Figure 1, except that wortmannin (200 nM or 10 μM) or quercetin (20 μM) were added at hour 3. **(A)** Flow cytometry was used to calculate the ratio of PM localized EGFR fluorescence to total EGFR fluorescence, and this ratio for each time point ± wortmannin is plotted. Error bars indicate ± SE of five independent experiments in which approximately 8,000 EGFR-GFP expressing cells were analyzed at each time point. * p < 0.05; *** p < 0.001 **(B)** Representative confocal images of RBL cells expressing EGFR-GFP either untreated or treated with 200 nM or 10 μM wortmannin from 8 hr time points quantified in **A**. **(C)** Flow cytometry was used to calculate the ratio of PM localized EGFR fluorescence to total EGFR fluorescence, and this ratio for each time point ± quercetin is plotted. Error bars indicate ± SE of four independent experiments in which approximately 8,000 EGFR-GFP expressing cells were analyzed at each time point. ** p < 0.01 **(D)** Representative confocal images of RBL cells expressing EGFR-GFP either untreated or treated with 20 μM quercetin from 8 hr time points quantified in **C**. Two different cells with 20 μM quercetin shown to represent the range of distributions observed. Scale bars show 5 μm.

Nanomolar concentrations of wortmannin have been shown to inhibit PI3-kinase (IC50 = 5 nM) [41], whereas significantly higher concentrations are needed to similarly inhibit PI 4-kinases [42,43]. We found that 10 μM wortmannin prevents EGFR-GFP from trafficking to the PM (Figure 7A), and this receptor is intracellularly retained (Figure 7B), with some localization in both the ER and the Golgi apparatus (Figure 3A,B). When cells are treated with 200 nM wortmannin, EGFR-GFP biosynthetic trafficking to the PM is somewhat diminished (Figure 7A); but still readily detectable there (Figure 7B), indicating that inhibition of PI3-kinase is not sufficient to prevent EGFR-GFP trafficking to the PM. Quercetin has been shown to generally inhibit kinases, including PI kinases, by competition with ATP for the active site [44]. Quercetin significantly reduces biosynthetic trafficking of EGFR (Figure 7C), and only a small percentage of EGFR-GFP is detected at the PM after 8 hr when cells are treated with 20 μM quercetin (Figure 7C,D). As with wortmannin, inhibition by quercetin results in substantial accumulation of EGFR in the Golgi apparatus, suggesting that these PI kinase inhibitors are less effective at inhibiting ER to Golgi trafficking than PAO. None-the-less, inhibition of biosynthetic trafficking by wortmannin and quercetin (Figure 7) is consistent with the conclusion that inhibition of trafficking with PAO (Figure 4) is primarily due to the inhibition of a PI kinase, rather than inhibition of tyrosine phosphatases.

**Figure 8:**
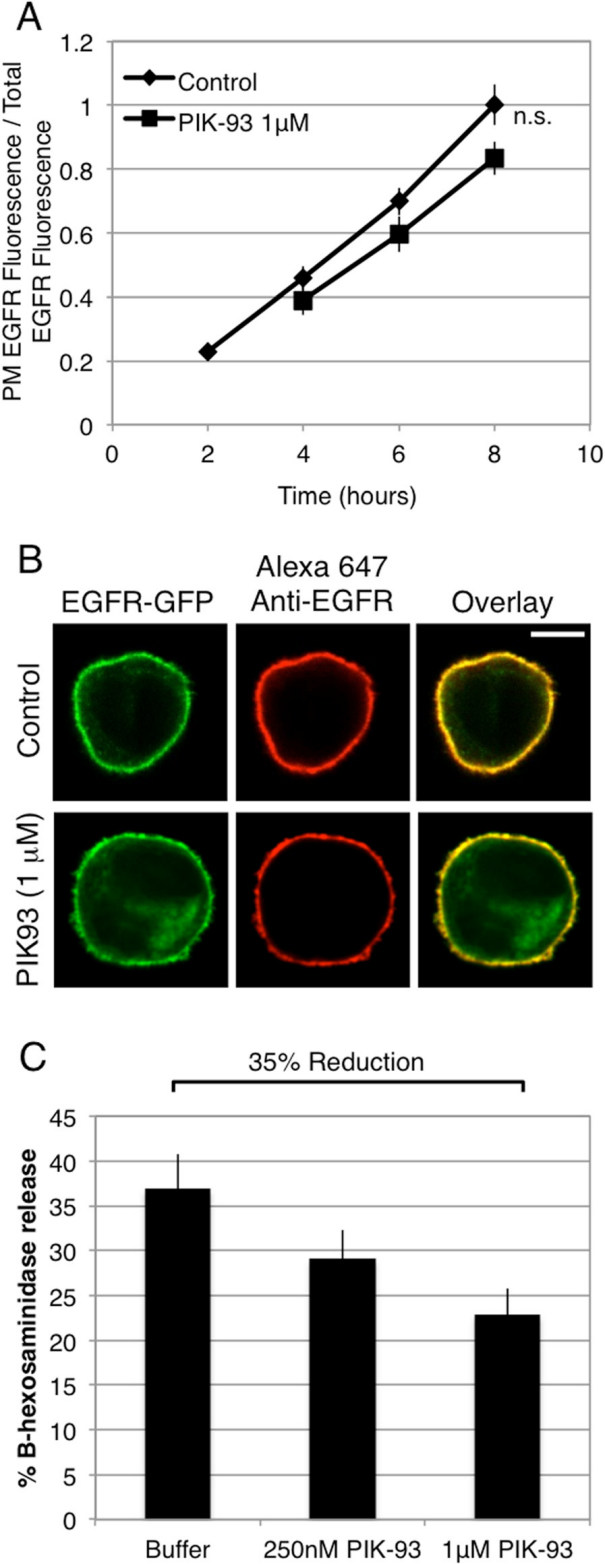
**Treatment with PIK-93 does not significantly affect the biosynthetic trafficking of EGFR to the PM** ***.*** Live cells transiently expressing EGFR-GFP were processed as described for Figure 1, except that 1 μM PIK-93 was added to half of the cells at hour 3. **(A)** Flow cytometry was used to calculate the ratio of PM localized EGFR fluorescence to total EGFR fluorescence, and this ratio for each time point ± PIK-93 is plotted. Error bars indicate ± SE of five independent experiments in which approximately 8,000 EGFR-GFP expressing cells were analyzed at each time point. n.s., not significant **(B)** Representative confocal images of RBL cells expressing EGFR-GFP either untreated or treated with 1 μM PIK-93, cells are from 8 hr time points quantified in **A**. Scale bar shows 5 μm. **(C)** Degranulation was measured for cells pretreated with denoted concentrations of PIK-93 for 40 min before stimulation with antigen (10 μg/ml) for 25 min. Bars represent average degranulation response from two experiments, in which each condition was assayed in triplicate, error bars indicate ± SD.

Organelle-specific PI-kinases and phosphatases dictate distinct subcellular distributions of the individual PI species that control the timing and location of trafficking events [23]. Based on immunofluorescence, PI4KIIIα has been suggested to localize to the ER [22], but a more recent study indicates a largely cytoplasmic distribution [24]. Both of the type-II PI 4-kinases localize to the Golgi complex and endosomes, and PI4KIIIβ localizes more selectively to the Golgi complex [18]. To determine whether inhibition of a different isoform of PI4K similarly inhibits biosynthetic trafficking of EGFR from the ER to the Golgi apparatus, we tested the possible role of *cis*-Golgi localized PI4KIIIβ. PIK-93 (phenylthiazole) is a selective inhibitor of PI4KIIIβ (IC50 = 19 nM; [45]), and we found that this compound does not significantly affect the biosynthetic trafficking of EGFR-GFP, even when used at a concentration (1 μM) that should completely inhibit PI4KIIIβ (Figure 8A,B). To confirm its potency, we tested the effect of PIK-93 on degranulation of RBL cells in response to multivalent antigen, which has been shown to utilize PI4KIIIβ [46]. As shown in Figure 8C, we find that 1 μM PIK-93 reduces stimulated degranulation by approximately 35%, consistent with reduction in degranulation observed with PI4KIIIβ silencing [46].

## Discussion

Previous biosynthetic trafficking studies have frequently relied on the use of the temperature sensitive mutant of vesicular stomatitis virus glycoprotein (VSV-G^ts^), which can be synchronized to undergo ER to PM trafficking in a temperature-dependent manner [47]. The method we describe can be utilized to evaluate biosynthetic trafficking of a wide variety of transiently transfected proteins in different cell types without the necessity of pulse-chase radiolabeling and immunoprecipitation. Following transient transfection, cells are incubated overnight at a restrictive temperature (room temperature, 22°C, in the present study). During this incubation, protein synthesis occurs without subsequent protein trafficking out of the ER. The following day, when cells are shifted to 37°C, the synthesized protein moves through the biosynthetic pathway and its presence at the PM can be monitored over time (Figure 1).

Although incubation at 22°C has been reported to impair biosynthetic trafficking from the ER in some cell systems [29,30]; this temperature may not be universally applicable, as 15°C is more generally considered necessary to block biosynthetic trafficking at the level of the ER-exit sites and/or ERGIC [48,49]. This lower temperature may be an alternative in other applications. Additionally, EGFR is a PM localized protein that has a particularly long half-life of 8 to 24 hours depending on the cell type and level of expression. As shown in Figure 1, the presence of EGFR at the PM steadily increased from hours 2 through 10 and only after hour 10 does the ratio of PM localized EGFR fluorescence to total EGFR fluorescence begin to decrease. Thus, in the case of EGFR, endocytosis was not a contributing factor on the time scale used for the pharmacological experiments; however, this will not be the case for all potential proteins of interest. Therefore, prior to performing pharmacological or genetic manipulations one must determine the kinetics of the arrival of their protein of interest at the PM as well as the amount of time it is stably localized there, before endocytosis becomes a contributing factor.

Our flow cytometry-based method can be used to investigate a variety of means for intervention of biosynthetic trafficking, including molecular genetic manipulations. In the present study, we evaluated pharmacological effects. We first verified that this system is sensitive to of BFA, which is a well-documented inhibitor of this process (Figure 2). Following this validation of our method, we examined the trafficking of EGFR (Figures 1, 2, 3, 4, 5, 7, 8) and a model GPI-linked protein (Figure 6), both of which were fluorescently tagged and could be labeled with an extracellular epitope-binding antibody to visualize the PM-associated pool of protein. We used flow cytometry, complemented with confocal microscopy, to analyze the biosynthetic trafficking process. Flow cytometry is a convenient way to analyze thousands of positively transfected cells in a short amount of time and thus allows for a more rigorous statistical analysis of the data as compared to single cell imaging. Confocal microscopy provides representative images of our flow cytometry samples. The less flattened morphology of these suspended RBL cells permits clearer distinction of the PM than for more flattened cells; however, this morphology is more limited in terms of defining precise intracellular protein localizations. Regardless of the cell system, we note that, in the event that the PM-associated and total protein pools cannot be differentially labeled, biochemical methods, such as endoglycosidase H digestion assays [50], can also be used to quantify biosynthetic trafficking following this general approach, as demonstrated in the present study (Figure 5).

Of the different isoforms of PI 4-kinase, PI4KIIIα is preferentially inhibited by low micromolar concentrations of PAO [18,21]. We find that cells treated with PAO show a dose-dependent inhibition of biosynthetic trafficking of EGFR-GFP to the PM (Figure 4A,B). Nearly complete inhibition of trafficking is attained for cells treated with 2.5 μM PAO (Figure 4), and EGFR is retained in the ER (Figure 3). At this concentration, PAO causes strong inhibition of downstream IgE receptor signaling that correlates with inhibition of phosphoinositide synthesis in these RBL cells [34].

To test the possibility that inhibition of biosynthetic trafficking by PAO is due to the inhibition of enzymes other than PI4KIIIα, including tyrosine phosphatases [38], we analyzed the effects of two other PI 4-kinase inhibitors, wortmannin and quercetin [34], which do not inhibit tyrosine phosphatases. We observed dose-dependent inhibition of biosynthetic trafficking of EGFR-GFP with each (Figure 7), thus supporting the evidence that PAO treatment inhibits biosynthetic trafficking by inhibiting a PI 4-kinase, rather than a tyrosine phosphatase. Never the less, we cannot completely rule out other targets of PAO for its inhibitory effects, and future studies will require complementary tests of this mechanism using molecular genetic approaches.

Due to the predominant localization of the other three PI 4-kinase isoforms (IIα, IIβ, and IIIβ) to the Golgi, we hypothesize that a distinct pool of PI4P in the ER synthesized by PI4KIIIα is important for biosynthetic trafficking. This hypothesis is supported by our observation that inhibition of the Golgi-localized PI 4-kinase, PI4KIIIβ, does not significantly affect trafficking of EGFR to the PM (Figure 8). Although there is evidence that Golgi to PM trafficking also requires PI4P synthesized in the Golgi apparatus [23], we suggest that inhibition of one of the three Golgi-localized PI 4-kinases can be compensated by the other (Type II) PI4-kinases associated with this organelle.

What is the mechanism by which PI4P participates in the ER exit of PM-destined proteins? PI4P has been shown to support the reconstitution of yeast COPII-dependent budding from synthetic liposomes [15]. Consistent with this, PI4P has been shown to promote ER exit of VSV-G^ts^ by regulating ERES membrane budding and COPII coat nucleation in mammalian cells [16]. Appearance of PI4P at ERES is transient and coincident with cargo concentration in these sites [16]. We hypothesize that PI4P mediates its effects on the biosynthetic trafficking of proteins via electrostatic interactions with basic residues in protein sequences, located either in the cargo or in components of the trafficking machinery. For example, the crystal structure of Sec23-Sec24 dimer in the COPII coatmer complex exhibits a surface of basic residues on the membrane-facing side that may bind to PI4P [51].

Because we observe a primary accumulation of EGFR-GFP and GPI-YFP in the ER of RBL cells in which PI4KIIIα has been inhibited by PAO, it is likely that this accumulation is due to depletion of an ER-localized pool of PI4P necessary for exiting the ER. Alternatively, accumulation in the ER could result from depletion of a Golgi-localized pool of PI4P that results in retrograde trafficking to the ER. The localization of PI4KIIIα is complex: Mammalian PI4KIIIα is the homolog of yeast Stt4, a PM-associated protein [52] that has more recently been suggested to localize to specific regions of the PM called PI-kinase (PIK) patches, important for signaling [53]. Paradoxically, although Stt4 is not commonly detected in the ER in yeast, the PI4P pool generated by this kinase is mainly dephosphorylated by the ER-localized phosphoinositide phosphatase, Sac1p [54]. Additionally, PI4KIIIα contains a FFAT (two phenylalanines [FF] in an acidic track) motif that binds to the integral ER proteins Scs2/22 in yeast, or VAP proteins in mammals [55], consistent with localization to the ER. In mammalian cells, PI4KIIIα was initially reported to localize primarily to the ER based on immunofluorescence [22]. Furthermore, it has been demonstrated that silencing of PI4KIIIα by siRNA results in a large reduction in the number of ERES, which in turn correlates with decreased ER-to-Golgi transport [56]. However, PI4KIIIα also has been reported to be responsible for the generation of hormone-sensitive phosphoinositide pools in the PM [25], and a recent report describes a predominantly cytoplasmic localized PI4KIIIα distribution with some PM association that contributes to PM identity [24].

Future work to visualize and selectively deplete PI4P either in the ER or the Golgi apparatus may lead to better understanding of the roles of the pools of PI4P in each of these organelles that are important for biosynthetic trafficking. The Sac1 phosphatase cycles between the ER and Golgi apparatus and is a key regulator of PI4P in mammalian cells [57]. Szentpetery et al. [27] recently described a drug-inducible molecular approach to specifically target the effects of Sac1 phosphatase activity to a particular intracellular membrane. Use of this technique to selectively deplete PI4P either in the ER or the Golgi apparatus may lead to better understanding of the origins of the pools of PI4P important for biosynthetic trafficking. While the localization of PI4P to the Golgi has been extensively reported, new tools to visualize PI4P pools in cells have revealed pools of this lipid associated with the PM and late endosomes/lysosomes [58]. It will be interesting and informative to test whether these probes are capable of recognizing an ER-associated pool of PI4P in this cell system. Alternatively, further support for the necessity of PI4KIIIα activity, as implicated by our pharmacological approach, could be provided with the use of siRNA to genetically silence this protein. The flow cytometry based method we have developed will continue to be useful in evaluating these more molecular approaches.

## Conclusions

Biosynthetic trafficking of receptors and other membrane proteins to the PM underlies the capacity of these proteins to participate in crucial cellular functions. Elucidating the mechanisms of the trafficking process is fundamental to understanding and interfering with the cellular responses that PM-localized receptors regulate. Toward this end, we developed a novel flow cytometry-based method to examine the biosynthetic trafficking of transiently transfected proteins. We utilized this method to show that PI4P is important for biosynthetic trafficking of both a transmembrane and a GPI-linked protein to the PM. In particular, we took advantage of a number of well-characterized inhibitors of the different PI 4-kinase isoforms [18] to provide pharmacological evidence that the type IIIα isoform of PI4K may be responsible for synthesizing the relevant pool of PI4P. This simple, flow-cytometry based biosynthetic trafficking method could be widely applicable to different classes of proteins and inhibitors using pharmacological and molecular approaches.

## Methods

### Materials and expression plasmids

All cell culture reagents and all Alexa-dye conjugated secondary antibodies were from Invitrogen. The anti-N-terminal human EGFR antibody (clone LA1) used for flow cytometry and immunochemistry, and the anti-C-terminal human EGFR antibody (clone E235) used for immunochemistry were from Millipore Corp. Anti-protein disulfide isomerase (PDI) mAb was from Affinity Bioreagents, and anti-GM130 was from BD Biosciences. PIK-93 was from Symansis; wortmannin and brefeldin-A were from Calbiochem. Anti-GFP mAb, phenylarsine oxide (PAO), quercetin, as well as any chemical not noted otherwise were purchased from Sigma-Aldrich Chemical Co. The human EGFR-GFP construct was a gift from Dr. J. Koland (U. Iowa) and has been described previously [59]. YFP-GPI, a gift from Dr. T. Baumgart (U. Penn.) is a GPI-anchored protein containing yellow fluorescent protein and a consensus *N*-glycosylation site fused to the GPI-attachment signal of lymphocyte-function-associated antigen 3 (LFA-3) [60].

### Cell culture

RBL-2H3 mast cells were grown in MEM containing 20% (v/v) fetal bovine serum (FBS) (Atlanta Biologicals) and 10 μg/ml gentamicin sulfate as described previously [61]. In preparation for imaging or flow cytometry, cells were plated at 25-50% confluence in 35 mm coverslip wells (MatTek Corporation) or 60 mm dishes, respectively. After approximately 20 h, the RBL cells were transfected with EGFR-GFP or YFP-GPI using Fugene HD (Promega) per manufacturers’ instructions, with modification to enhance transfection efficiency in the RBL cells previously described [61].

### Biosynthetic trafficking method

On day one, approximately 1x10^6^ RBL cells were plated in 60 mm dishes or 3.5x10^5^ RBL cells were plated in 35 mm coverslip dishes. On day two, cells were transfected using Fugene HD as described above. Cells were allowed to recover from transfection for 30 min to 1 hr at 37°C in culture medium. Subsequently, the medium was buffered with 40 mM HEPES, and the dishes were sealed with parafilm and incubated at room temperature in the dark for 12 to 14 hr. On day three, cells were returned to regular culture medium and placed at 37°C; this point is time zero in the time course. For most experiments, pharmacological agents were diluted in buffered saline solution (BSS: 20 mM HEPES, 135 mM NaCl, 1.8 mM CaCl_2_, 2 mM MgCl_2_, 5.6 mM glucose, 1 mg/ml BSA, pH 7.4) and added at hour 3 of the time course (for control samples, culture medium was replaced with BSS at this time in parallel). Samples from each time point were processed for confocal fluorescence microscopy or flow cytometry as described below.

### Confocal fluorescence microscopy

RBL cells were washed once with BSS, fixed using 4% paraformaldehyde with 0.1% glutaraldehyde, permeabilized (or not) with 1% v/v Triton X-100, and labeled for 1 hour with specified antibodies in phosphate buffered saline (PBS) with 10 mg/ml BSA and 0.01% w/v sodium azide (PBS/BSA). Images were collected using an upright Leica TCS SP2 laser scanning confocal microscope (Leica Microsystems, Exton, PA) with a 63 x 0.9 NA, HCX APO L U-V-I water-immersion objective.

### Flow cytometry

Herein, RBL cells were harvested by trypsinization; however, commercial non-enzymatic dissociation reagents or PBS/EDTA could be similarly used if there is concern trypsin could harm the protein of interest. Following dissociation, cells were washed once with BSS, fixed using 4% paraformaldehyde with 0.1% glutaraldehyde, and quenched with PBS/BSA. Cells expressing EGFR-EGFP or YFP-GPI constructs were labeled with appropriate antibodies for 1 hr in PBS/BSA. Samples were evaluated using a Becton Dickinson (BD) LSR II flow cytometer, and data were analyzed using BD FACSDiva software. Analysis was gated to include single cells on the basis of forward and side light-scatter, and data from single-color samples were used to determine the gates for positive fluorescence from each fluorophore. The ratio of PM-associated EGFR fluorescence (Alexa 647 antibody labeled) to total EGFR-GFP fluorescence was normalized to the 7 or 8 hr time point for each timecourse shown.

### EndoH sensitivity assay

To generate whole cell lysates, RBL cells were washed in PBS, incubated in lysis buffer (25 mM Tris, pH 7.4, 100 mM NaCl, 1 mM EDTA, 1% (v/v) Triton 100, 1 mM DTT, 1 mM sodium orthovanadate, 1 mM β-glycerol phosphate, 1 μg/ml leupeptin, and 1 μg/ml aprotinin), and supernatants were retained following microcentrifuge centrifugation. Protein concentrations of the whole cell lysates were determined using the Bio-Rad DC protein assay. Whole-cell lysates were divided into three equal portions that then were untreated, or treated with EndoH or PNGase F according to the manufacturer’s instructions (New England Biolabs). Samples were incubated for 24 h at 37°C, and the digestion reactions were stopped by heating at 95°C for 5 min after the addition of SDS-PAGE sample buffer. Samples were analyzed by immunoblotting.

### Degranulation: β-Hexosaminidase Release

Cells were plated in triplicate at a density of 5x10^5^/well and incubated overnight in the presence of 1 μg/ml anti-DNP IgE. The next day, cells were treated with PIK-93 (250 nM or 1 μM) for 40 min in BSS without BSA, and β-hexosaminidase release in response to DNP-BSA was assessed as described previously [62].

### Data analysis

Statistical analyses were determined using GraphPad Prism (GraphPad Software, La Jolla, CA) using Student’s *t* test, with p < 0.05 considered statistically significant.

## Abbreviations

BSS: Buffered saline solution
BFA: Brefeldin A
DAG: Diacylglycerol
EGFR: Epidermal growth factor receptor
ER: Endoplasmic reticulum
ERES: ER exit site
ERGIC: ER-Golgi intermediate compartment
GPI: Glycophosphatidylinositol
IP_3_: Inositol 1,4,5-trisphosphate
PAO: Phenylarsine oxide
PBS: Phosphate buffered saline
PDI: Protein disulfide isomerase
PI4P: Phosphatidylinositiol-4-phosphate
PI(4,5)P_2_: Phosphatidylinositol 4,5 bisphosphate
PIP_3_: Phosphatidylinositol 3,4,5-trisphosphate
PM: Plasma membrane
RBL: Rat basophilic leukemia
